# Carbon Quantum Dots Based on Marine Polysaccharides: Types, Synthesis, and Applications

**DOI:** 10.3390/md21060338

**Published:** 2023-05-31

**Authors:** Fernando G. Torres, Karen N. Gonzales, Omar P. Troncoso, Victoria S. Cañedo

**Affiliations:** Department of Mechanical Engineering, Pontificia Universidad Católica del Perú, Av. Universitaria 1801, Lima 15088, Peru

**Keywords:** carbon quantum dots, marine polysaccharides, drug delivery, biosensing, bioimaging

## Abstract

The marine environment offers a vast array of resources, including plants, animals, and microorganisms, that can be utilized to extract polysaccharides such as alginate, carrageenan, chitin, chitosan, agarose, ulvan, porphyra, and many more. These polysaccharides found in marine environments can serve as carbon-rich precursors for synthesizing carbon quantum dots (CQDs). Marine polysaccharides have a distinct advantage over other CQD precursors because they contain multiple heteroatoms, including nitrogen (N), sulfur (S), and oxygen (O). The surface of CQDs can be naturally doped, reducing the need for excessive use of chemical reagents and promoting green methods. The present review highlights the processing methods used to synthesize CQDs from marine polysaccharide precursors. These can be classified according to their biological origin as being derived from algae, crustaceans, or fish. CQDs can be synthesized to exhibit exceptional optical properties, including high fluorescence emission, absorbance, quenching, and quantum yield. CQDs’ structural, morphological, and optical properties can be adjusted by utilizing multi-heteroatom precursors. Moreover, owing to their biocompatibility and low toxicity, CQDs obtained from marine polysaccharides have potential applications in various fields, including biomedicine (e.g., drug delivery, bioimaging, and biosensing), photocatalysis, water quality monitoring, and the food industry. Using marine polysaccharides to produce carbon quantum dots (CQDs) enables the transformation of renewable sources into a cutting-edge technological product. This review can provide fundamental insights for the development of novel nanomaterials derived from natural marine sources.

## 1. Introduction

In recent years, research in the area of nanomaterials has increased due to their essential role in the development of technologies related to food, agriculture, energy, and medicine [1,2,3,4,5]. Biopolymer nanoparticles have been used in different fields of medicine and healthcare [6,7], including drug delivery systems [8], bioimaging [9], and biosensing [10]. Other applications of biopolymer nanoparticles include fluorescent biosensors [11], wastewater treatment [12], edible films [13], and packaging materials [14].

Biopolymers are environmentally friendly and have unique biocompatibility, biodegradability, and non-toxicity properties. They can be obtained from natural sources such as plants, animals, microorganisms, and agricultural wastes [15]. Organisms from marine ecosystems also represent an important source for polysaccharide extraction. For instance, carrageenan, sodium alginate, ulvan, agarose, chitosan, chitin, and other polysaccharides are extracted from marine algae, marine crustaceans, fish, and microorganisms [16]. Marine polysaccharides are abundant, low-cost, non-toxic, biodegradable, and biocompatible. They have been used for the development of novel nanomaterials including nanofibers [17], nanoparticles [18], nanocrystals, nanogels [19], as well as carbon quantum dots [1].

Quantum dots are nanoparticles featuring fluorescence activity; i.e., they emit light of specific wavelengths after absorbing an initial radiation. This fluorescence activity is evidence of the quantum confinement effect that may take place in nano-sized systems. Traditionally, quantum dots were synthesized from heavy metals such as Cd and Pb. However, quantum dots can also be obtained from carbon. Carbon quantum dots (CQDs) were first prepared during the purification of single-wall carbon nanotubes (SWCNs) [20]. These CQDs can be synthesized from organic materials and exhibit excellent water solubility, antibacterial properties, selectivity, and sensitivity, which makes them suitable for applications in many fields, including bioimaging, biosensing, cancer therapy [21], gene delivery [22], drug delivery, metal ion detection, and water treatment [23,24,25,26,27]. CQDs feature robust and tunable fluorescence properties, which make them suitable for the fabrication of optoelectronic devices [28], sensors [29], photocatalytic solar cells [30], and even smart packaging films [31].

CQDs are usually synthesized from organic materials. They are mainly formed by C, H, and O. Several authors have modified the surface of these CQDs with the addition of different types of heteroatoms such as N, S, and P to obtain a variety of heteroatom-doped CQDs (N-CQDs, S-CQDs, and P-CQDs). Doped CQDs feature an increase in the fluorescence emission intensity and exhibit a colorful luminescence since the emission wavelength shifts to the near-infrared or blue regions as compared to non-doped CQDs. 

CQDs can be synthesized by different methods that use a great variety of precursors as raw materials, including small molecules such as citric acid, glucose, amino acids, diamine, and phenylenediamine [32,33] and biopolymers extracted from natural sources such as chia [34], crown flower (*Calotropis gigantea*) [12], silk (*Bombyx mori*) [35], tapioca [36], and zein [37], among others. Marine polysaccharides have also been reported to be used as a carbon source for the synthesis of CQDs. Most importantly, the great content of heteroatoms such as N and S in marine polysaccharides makes them especially useful for the preparation of naturally doped CQDs. In contrast to land polysaccharides, many marine polysaccharides have been shown to be sustainable precursors for sulfur- and nitrogen-containing carbon nanomaterials. In fact, a high number of marine polysaccharides are decorated with sulfate ester groups, including carrageenans, agarans, fucoidans, and ulvans, among others [38]. Carrageenan is one of the most important sulfated polysaccharides. It features a content of about 25–39 wt.% of ester sulfate. Another important marine polysaccharide is chitosan, which is rich in nitrogen content (7 wt.%) and has been used in the preparation of N-doped carbon nanomaterials [39]. Doping is one of the most effective treatments used to tune the properties of QCDs. The doping of heteroatoms can manipulate the photoluminescence properties of QCDs in both the intrinsic and surface electronic structures. This changes the amount of energy needed for the photoexcitation process to take place. The effect of dopants will be further discussed in Section 2.

The methods most widely used for the preparation of CQDs are hydrothermal and microwave carbonization. These facile and low-cost procedures allow for the preparation of uniform-sized CQDs. During these processes, N-self-doping, S-self-doping, and N-S-self-doping can occur in the carbon skeleton. Heteroatom doping on CQDs enables significant changes in optical and surface properties. 

The present review will focus on the recent progress regarding carbon quantum dots (CQDs) based on marine polysaccharides such as chitosan, chitin, carrageenan, alginate, porphyra, agarose, chondroitin sulfate, and ulvan. As CQDs have outstanding optical properties, understanding the primary mechanisms related to their fluorescence emission, absorbance, quenching, and quantum yield is highly important. We also highlight the processing routes, properties, and classified CQDs used in applications related to biosensing, bioimaging, drug delivery, fluorescence sensors, film packaging, and others. In addition, we place a particular emphasis on the natural heteroatom doping of the CQDs produced from marine polysaccharides, which improves their optical and surface properties. This review can provide fundamental insights for the development of novel nanomaterials based on marine polysaccharides from various natural sources.

## 2. Optical Properties of Carbon Quantum Dots (CQDs)

CQDs are nanostructures in which the movement of electrons in the three directions of Euclidean space is confined. This phenomenon is known as the quantum confinement effect and takes place when the particle size approaches the Bohr radius. The quantum confinement effect produces the discretization of the energy levels (i.e., Fermi energy levels) due to the modification of the electronic structure. This discretization of energy forms bands: the valence and the conduction bands, which are separated by a finite energy gap called the “bandgap”. The size of the bandgap depends on the size of the particles. That is, if the size of the particles decreases, the size of the bandgap increases. The larger the bandgap size, the more energy is required to bring the electron from its ground state to an excited state. This determines the amount of energy emitted as the electron descends from the excited state to the ground state. That is, the more energy absorbed, the more energy emitted. For this reason, the size of the bandgap influences the optical properties of the CQDs described in this section.

### 2.1. Absorbance Properties

**Figure 1:**
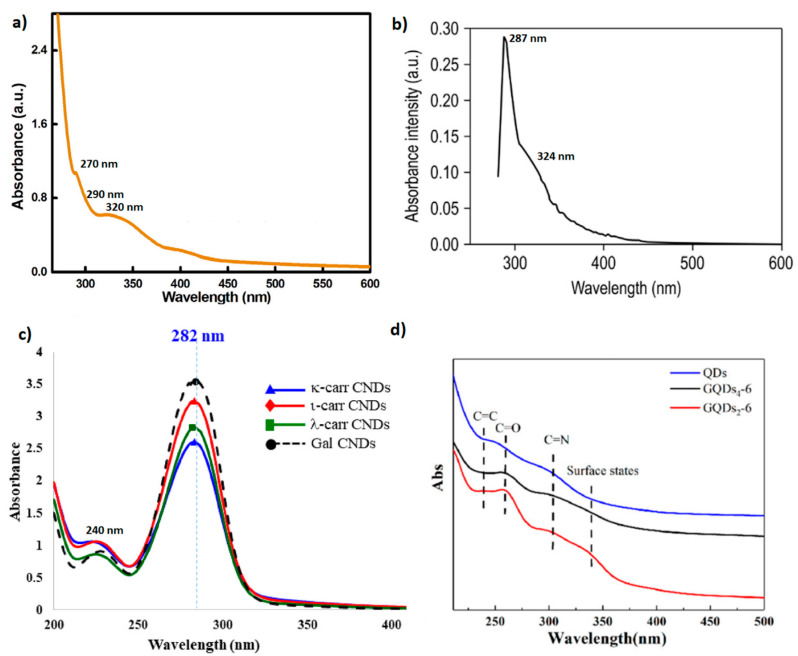
UV–vis absorption spectra of (**a**) CQDs based on agarose; (**b**) chondroitin sulfate; (**c**) carrageenan; (**d**) chitosan. Reproduced with permissions from (**a**) [40]; (**b**) [41]; (**c**) [42]; (**d**) [43].

Absorbance refers to the absorption of external light in the form of a photon. If the energy of such a photon matches the difference between the energy levels of the excited and ground state, the electron jumps to a higher energy level. When carbon dots are exposed to radiation, they exhibit optical absorption peaks due to electronic transitions. Figure 1 shows UV–vis absorption spectra of different marine polysaccharide-derived CQDs. The intrinsic absorption of π-π* transition is related to C=C and –C–C– bonds present in the sp2 carbon electrons in the core structure of the CQDs. It can be seen that the peak related to π-π* transition can be led to a red shift of the wavelength depending on the CQDs precursors. For example, chondroitin sulfate, carrageenan, chitosan, and agarose show a peak at 230 nm, 240 nm, 270 nm, and 287 nm, respectively.

Additional peaks can appear depending on the functional groups present on the exterior surface of CQDs. The UV–vis spectra (Figure 1b,c) of CQDs derived from chondroitin sulfate and carrageenan depict the n-π* transition attributed to C=O bonds and oxygen-containing compounds at about 324 nm and 282 nm, whereas the absorption of n-π* transition in agarose-derived CQDs (Figure 1a) is associated with amine (–NH_2_) at 290 nm and carboxylic group (–COOH) at 320 nm. Wu et al. (2022) reported that the n-π* transition for chitosan-derived CQDs is also associated with –C=N at 310 nm and multiple surface defect states in the 320–350 nm range. The above indicates that the optical properties of CQDs are influenced by the functional groups present on their surface.

### 2.2. Photoluminescence Properties

Photoluminescence is the emission of light (photon) caused by incident external energy in the form of a photon. The absorption of this photon excites an electron in the valence band, which moves into the conduction band. This phenomenon occurs if the photon’s energy equals or exceeds the bandgap energy. The excited electron returns to the ground state, releasing energy in the form of light (photons). There are two types of light emission: fluorescence and phosphorescence. Fluorescence occurs very shortly after photoexcitation, whereas phosphorescence continues long after photoexcitation.

**Figure 2:**
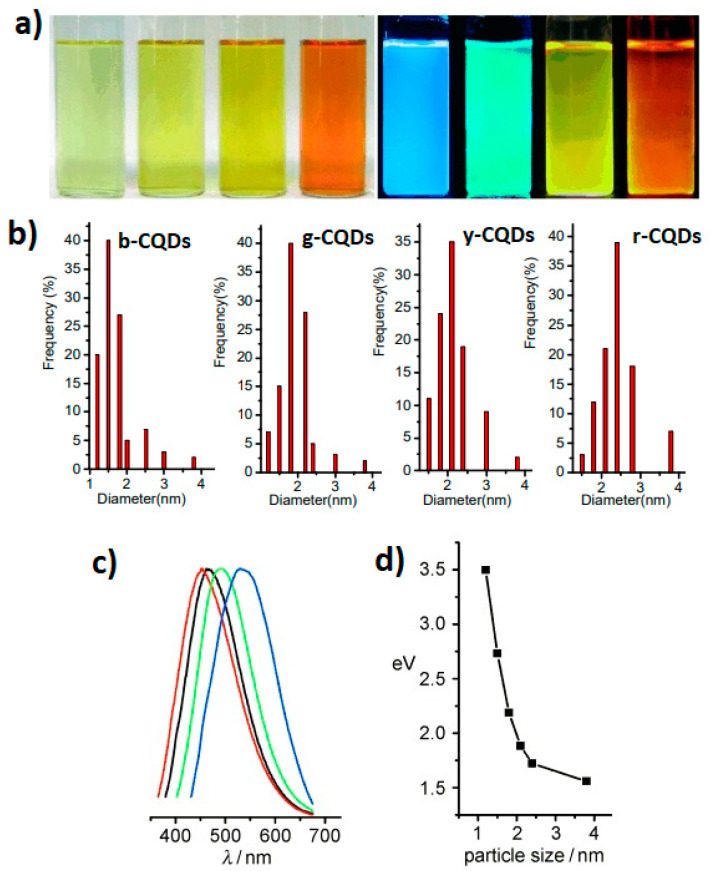
Photographs of (**a**) an aqueous dispersion of CQDs of different sizes under daylight (left) and UV light (right); (**b**) the size distribution for blue-, green-, yellow-, and red-emitting CQDs; (**c**) photoluminescence (PL) spectra of the four sizes of CQDs: the red, black, green, and blue lines are the PL spectra for blue-, green-, yellow-, and red-emitting CQDs, respectively; (**d**) band gap (eV) as a function of CQDs size. Reproduced with permissions from [44].

The light emitted by CQDs depends on the size of the quantum dots (Figure 2). As the size of the CQDs decreases, the bandgap increases, which means that the energy required to excite the electron will be higher than the energy emitted. For example, Figure 2 depicts that the small graphite-based CQDs (average 1.2 nm), medium CQDs (1.5 nm–3 nm), and large CQDs (3.8 nm) give UV light, visible light, and near-infrared emission [44]. The photoluminescence spectra of the four-sized blue-, green-, yellow-, and red-emitting CQDs are shown in Figure 2c. In Figure 2d, band gap energies are presented as a function of the four sizes of CQDs. It can be seen that the energy gap increases with a decrease in the CQDs’ size [44].

Dopant heteroatoms can also alter the photoluminescence characteristics of QCDs. Dopants act as substitutional guests with different valences and modify the electronic states of the host material. Common dopants used with marine polysaccharide-based QCDs are sulfur (S) and nitrogen (N). These dopants introduce an additional energy state within the energy gap between the highest occupied molecular orbital (HOMO) and the lowest unoccupied molecular orbital (HOMO–LUMO gap). This tends to result in a red-shifted spectrum due to the electronic donor effect of nitrogen and sulfur, resulting in lower energy for photoexcitation [45].

**Figure 3:**
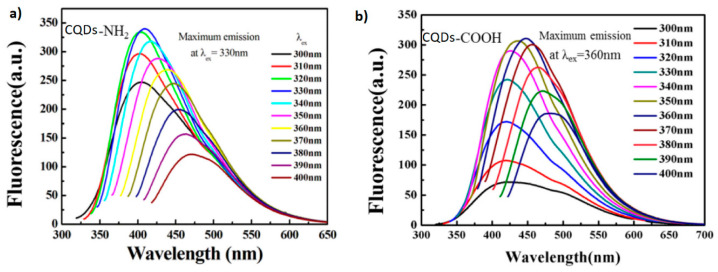
Photoluminescence spectrum of QCDs synthesized from (**a**) chitosan-acrylamide (CQDs-NH_2_) and (**b**) chitosan-acrylic acid (CQDs-COOH) excited at a range of wavelengths. Reproduced with permissions from [46].

The emission wavelength can also be tuned as a function of the excitation wavelength and the functional groups on the surface of the CQDs. Figure 3 shows the photoluminescence spectrum of QCDs synthesized from chitosan-acrylamide (CQDs-NH_2_) and chitosan-acrylic acid (CQDs-COOH). The emission wavelength (color of the light emitted) depends on the incident wavelength [46]. The fluorescence spectra of CQDs-NH_2_ and CQDs-COOH showed a maximum emission with a peak at 409 nm and 448 nm, respectively, when using excitation wavelengths of 330 nm and 360 nm.

### 2.3. Quenching Mechanism

Quenching is a process in which the intensity of emitted light (fluorescence) decreases completely due to the interaction between a fluorophore (i.e., a molecule with fluorescence properties) and other molecules or metal ions (i.e., Fe^2+^, Fe^3+^, and Cu^2+^, among others) [47]. There are two types of fluorescence-quenching mechanisms: static and dynamic quenching, which can be determined by measuring the fluorescence lifetime. Static quenching occurs when a non-fluorescent complex is formed between a fluorophore and a quencher in the ground state. The photoluminescence lifetime of the fluorophore remains constant as a function of the concentration of the quencher (e.g., metal ions). For instance, when Cu^2+^ is added to a phosphorus-doped carbon quantum dots (P-CQDs) solution, fluorescence quenching occurs because the functional groups on the surface of carbon dots act as electron donors. In contrast, copper ions act as electron acceptors, forming P-CQDs–Cu^+2^ complexes [48]. These cupric complexes generate a shift in the emitted wavelength. 

The quencher interacts with the fluorophore in an excited state in the dynamic quenching mechanism, and the photoluminescence lifetime is variable. For example, κ-Carrageenan-derived CQDs were used as a sensing probe to detect Fe^3+^ ions [49]. The fluorescence decay curves of κ-Carrageenan-derived CQDs with and without Fe^3+^ ions were evaluated. The average lifetime of κ-Carrageenan-derived CQDs decreased from 4.40 ns to 3.04 ns after adding Fe^3+^ ions. The change observed in the lifetime of the CQDs could be attributed to the electron transfers from excited κ-Carrageenan-derived CQDs to half-filled 3d orbitals of Fe^3+^ ions, leading to the fluorescence of the CQDs turning off.

### 2.4. Quantum Yield

The quantum yield (QY) is defined as the efficiency of converting absorbed light into emitted light (fluorescence). It is calculated as the ratio between the number of emitted and absorbed photons [50]. The quantum yield of CQDs is calculated using Equation (1), considering quinine sulfate (QS) as a reference material with a high quantum yield (54%) in sulfuric acid [51].

(1) $${QY}_{CQDs}={QY}_{QS}\times \left[\frac{I_{CQDs}}{I_{QS}}\right]\times \left[\frac{A_{QS}}{A_{CQDs}}\right]\times {\left[\frac{n_{CQDs}}{n_{QS}}\right]}^{2}\times 100\%$$

where “*I*” stands for the measure of the integrated photoluminescence (PL) intensities (emission wavelengths) at a specified excitation wavelength, “*A*” refers to the UV–vis absorbance or optical density, and “*n*” is the refractive index.

## 3. Marine Polysaccharides Precursors for the Synthesis of CQDs

Marine polysaccharides are environmentally friendly materials that can be used as precursors for synthesizing novel carbon dots. The most common marine polysaccharides used include chitosan, chitin, carrageenan, ulvan, alginate, agarose, and porphyra. The different marine polysaccharides used as a precursor can be classified according to their biological origin, such as polysaccharides from marine algae, crustacean exoskeletons, and fish. Table 1 summarizes the various types of marine polysaccharides utilized as precursors and the primary properties reported for the synthesized CQDs.

### 3.1. Polysaccharides Extracted from Marine Algae

#### 3.1.1. Carrageenan

Carrageenan is a linear sulfated polysaccharide extracted from red algae. There are three types of carrageenan, i.e., κ-Carrageenan, ɩ-carrageenan, and λ-carrageenan. The difference between these types of carrageenan is the number and position of ester-sulfate groups. In addition to the presence of ester-sulfate groups, carrageenan includes hydroxyl (–OH) groups. These groups can improve the water solubility and quantum yield of CQDs by introducing heteroatoms (oxygen and sulfur) on the surface of CQDs [49]. 

**Figure 4:**
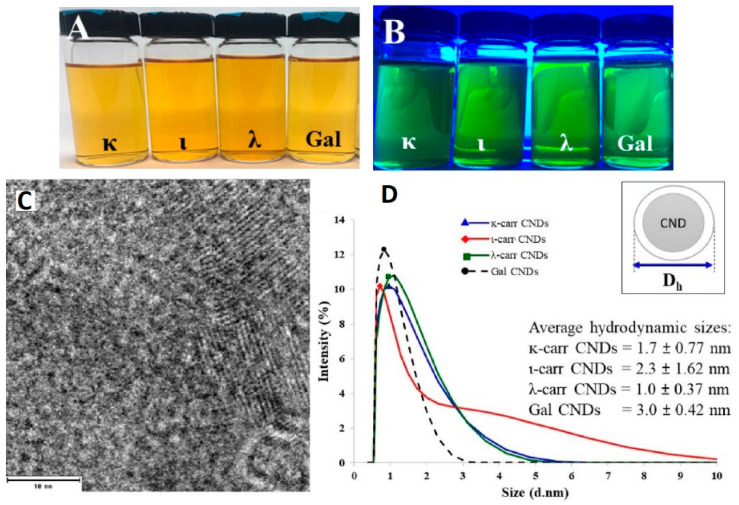
Photographs of (**A**) к-, ɩ-, and λ- carrageenan and galactose CQDs in water under daylight and (**B**) UV light; (**C**) representative TEM image of κ-Carrageenan CQDs, scale bar: 10 nm; (**D**) hydrodynamic size of as-prepared CQDs. Reproduced with permissions from [42].

Emma H. and Ahmed H. [52] used k-carrageenan to synthesize CQDs with a diameter of 2.1 nm. Other authors have reported that CQDs prepared with κ-Carrageenan had diameters ranging from 0.93 nm to 30 nm and quantum yield values reaching up to 69.27% [53] (Table 1). Leuterio et al. [42] used (к-, ɩ-, and λ-) carrageenan and galactose as carbon precursors for synthesizing CQDs. The carrageenans and galactose CQDs suspension in water showed a yellow color under daylight, while a luminous green color appeared under UV irradiation (Figure 4A,B). The morphology and size of the as-prepared CQDs were studied by TEM and dynamic light scattering (DLS), as shown in Figure 4. Figure 3c depicts quasi-spherical shapes, and the hydrodynamic sizes of the carrageenans and galactose CQDs are in the range of 1.7 nm–3 nm (Figure 4D). The authors proved that the sulfate content in carrageenan CQDs plays a critical role in their acidity properties. The starting materials к-, ɩ-, and λ- carrageenan have one, two, and three sulfated groups per disaccharide, in contrast to galactose, which has the same sugar structure as carrageenan but no ester sulfate groups. The pH values of к-, ɩ-, and λ-carrageenan CQDs and galactose CQDs suspension in water were 2.34, 2.22, 1.86, and 3.08, respectively. This result indicates that the degree of sulfation in CQDs plays a role in the final acidity properties.

Das et al. (2018) [53] synthesized nitrogen and sulfur co-doped CQDs (NSCQDs) using κ-Carrageenan and urea as a precursor and passivating agent, respectively. The average particle size of the NSCQDs was 3.5 nm, with a quasi-spherical and amorphous nature. The fluorescence lifetime and quantum yield of the NSCQDs were 4.06 ns and 69.27%, respectively. The stability of the aqueous dispersion of NSCQDs was evaluated via fluorescence intensities over two months. No alteration was detected at 360 nm excitation, and no precipitation was observed. In another study, Das et al. (2019) [54] reported CQDs with a two-centered emission using κ-Carrageenan with lemon juice as sulfur and carbon sources and benzalkonium chloride as the quaternizing agent. The obtained KLBC dots had a spherical and monodisperse form with an average size of 4.53 nm. The fluorescence properties revealed that the KLBC dots, with a quantum yield of 62.54%, had two fluorescence lifetimes of 7.47 ns and 5.79 ns at 295 nm and 375 nm excitation, respectively. The stability of the aqueous solution of the KLBC dots related to its fluorescence activity lasted for more than 90 days. 

Supchocksoonthorn et al. (2021) [55] used ɩ-carrageenan as a precursor to synthesize CQDs with an average size of 3.6 nm and a quasi-spherical shape. The ɩ-carrageenan CQDs had a high value of zeta potential (−45.5 mV), which enhanced the solubility of the CQDs in an aqueous solution. This is attributed to the presence of oxygen and sulfate ions.

#### 3.1.2. Agarose

Agarose is a linear polysaccharide derivative of agar, which is extracted from red algae. One of the common uses of agarose in laboratories and industry is in the form of a gel. Agarose gels are used in biological tests, including antimicrobial, antifungal, and electrophoresis. At the end of these assays, agarose gel residues are generated. This can be considered an environmental issue due to its potential contamination with bacteria, viruses, or mutagenic and carcinogenic chemicals. In this context, Chauhan et al. (2020) [40] synthesized, for the first time, CQDs using agarose waste as a carbon precursor. The size of the as-prepared CQDs was distributed in the range from 2 to 10 nm, with a distorted spherical shape. The optical properties of the CQDs achieved up to 62% quantum yield and revealed the presence of an sp^2^ hybridized graphitic core structure, according to the π-π* transition of –C=C– and –C–C–. Chauhan et al. pursued the development of CQDs from agarose waste to evaluate their use as fluorescent sensors [61,89]. These applications will be discussed further in Section 5.

#### 3.1.3. Sodium Alginate

Alginate is a linear biopolymer extracted from brown algae and some bacterial strains (*Azotobacter* sp. and *Pseudomonas* sp.). The chemical structure of alginate polysaccharide includes block sequences of α-ւ-guluronic acid and β-d-manuronic acid. Both units contain carboxyl and hydroxyl groups that can serve for sensing cations ions. They can even be chemically modified to tune their final characteristics. As an example, to enhance the fluorescent performance of CQDs derived from sodium alginate, Sun et al. (2020) [58] used glutaraldehyde as a cross-linking agent to modify sodium-alginate chains through the acetylation of aldehyde and hydroxyl groups. The acetylation treatment allows an increase in the value of the quantum yield of the as-prepared CQDs from 2.5% to 11%. The authors associated the increase in the fluorescence intensity with the restriction of non-radiative vibrational processes. This phenomenon results from the restriction of the vibration and rotation of subfluorophores (C=O, C–O), thus enhancing the fluorescence. 

**Figure 5:**
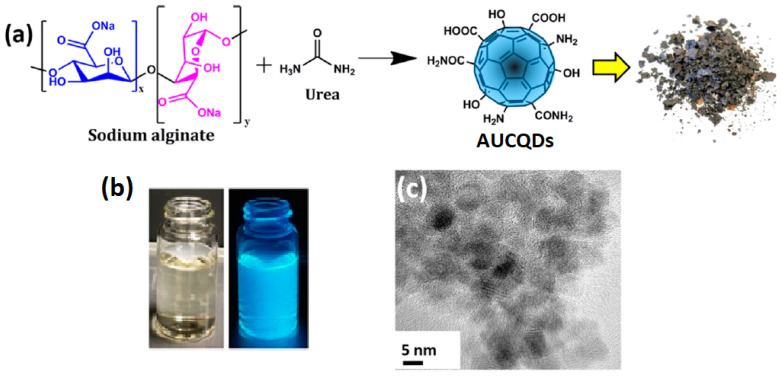
Schematic diagram for the synthesis of alginate-urea CQDs (AUCQDs) (**a**), AUCQDs dispersion in water under visible light (left) and UV light (right) (**b**), and TEM image of AUCQDs (**c**). Reproduced with permissions from [90].

Ganguly et al. (2020) [90] proposed modifying the surface carbon moieties of alginate CQDs by using urea. This approach allows for an increase in the number of hydrophilic groups (–OH, –COOH, and –CONH_2_) attached to the surface of the alginate CQDs (Figure 5). The average diameter of the as-prepared alginate-urea CQDs (AUCQDs) was greater (5.6 nm) than other alginate CQDs reported by Sun et al. (2020) (2–5 nm) [58] and lesser compared to the CQDs reported by Zhou et al. [22]. According to Ganguly et al. (2020), the AUCQDs exhibited fluorescence stability for more than 9 months, with a high quantum yield of 48.7%. The alginate-urea CQDs dispersion in water was yellow and transparent under visible light and exhibited blue fluorescence under UV light, as shown in Figure 5b.

Alginate can also serve as an efficient carbon precursor to develop graphene quantum dots (GQDs). The resulting GQD, with 10–20 nm of length and 1–1.5 nm of thickness, exhibited blue emission (Atienzar et al. [59]). The authors also reported that the dimensions of the GQDs depend on the number of laser shots during the ablation process. Thus, GQDs with few layers were obtained after 40 min of ablation.

#### 3.1.4. Other CQDs Derived from Algae Polysaccharides

Fucoidan is a fucose-enriched and sulfated polysaccharide that can be isolated from brown algae. Recently, fucoidan was used as a carbon precursor for developing dark-brown CQDs powder (Tang et al. [62]). The average size of the fucoidan-derived CQDs (FCQDs) was 7.15 nm with a spherical shape. The aqueous solution of the FCQDs was yellow under sunlight, emitting green fluorescence under 365 nm UV light excitation. The authors reported that the FCQDs had a maximum excitation wavelength of 362 nm and a maximum emission wavelength of 453 nm. Since fucoidan is a sulfated polysaccharide, the sulfur content was measured to confirm the presence of SO_4_^2−^, and it was found to be 3.15%. Moreover, the zeta potentials of FCQDs reveal that these nanoparticles have a negative charge of −15.80 mV, which agrees with the S content. The sulfate content in fucoidan has been associated with its antibacterial activities on oral pathogenic bacteria such as *H. pyroli* and *S. mutans*. The antibacterial efficiency against *E. faecalis* in the presence of raw fucoidan and FCQDs was studied. Compared with raw fucoidan, the FCQDs showed a higher inhibition ratio (85%). This was associated with the size of FCQDs, which increases their bacterial permeability. In addition, sulfate groups damage bacterial cytoplasmic membranes and cause the dissolution of proteins and other essential molecules in bacteria.

Another algae-derived sulfated polysaccharide is porphyra, extracted from red algae. Porphyra comprises a linear sequence of alternating 3-linked β-D-galactopyranosyl and 4-linked α-ւ-galactosyl 6-sulfate units. Chen et al. (2017) reported the use of porphyra polysaccharide for the first time in developing CQDs [63]. These novel carbon precursors allowed for the production of PCQDs with spherical shapes ranging from of 1 to 9 nm. The optical properties of the PCQDs reveal that the quantum yield was 56.3%. The PCQDs had blue fluorescence under 365 nm UV light excitation, and the maximum excitation wavelength and emission wavelength appeared at 356 nm and 448 nm, respectively. Despite porphyra being a sulfated polysaccharide, the zeta potentials of PCQDs reveal that these nanodots have a positive charge of 23.54 mV. This result can be associated with the use of a surface passivation agent (ethylenediamine). The surface modification of PCQDs with ethylenediamine provided an efficient carrier for genes. 

In addition to red and brown algae, green algae from the genus Ulva have been proposed as a carbon-rich source for developing CQDs. Singh et al. (2018) used *U. lactuca*, an ulvan-rich polysaccharide source, to obtain CQDs through a carbonization process [64]. The quantum yield of the synthesized CQDs achieved an increase of up to 68% and showed a spherical shape in the range of 20–40 nm. The size reported could be associated with a lower carbonization temperature (90 °C) than other procedures for obtaining CQDs from algae sources.

### 3.2. Polysaccharides Extracted from the Exoskeletons of Crustaceans

#### 3.2.1. Chitin

Chitin, poly(β-(1-4)-N-acetyl-D-glucosamine, is an amino polysaccharide commonly extracted from seafood waste products (i.e., lobster, prawn, crayfish, and shrimp shells) [91,92,93]. The crustacean shell is treated with acid and alkaline reagents to remove minerals and proteins in order to obtain chitin. Other processing routes include the use of lactic acid bacteria and enzymes such as *Lactobacillus* sp. and proteinase K to obtain chitin [94,95].

The presence of nitrogen in chitin has driven an increasing interest in its use as a precursor for developing nitrogen-doped CQDs (NCQDs). According to Jiang et al. (2020), NCQDs can be synthesized by hydrothermal carbonization of chitin. It allows for the introduction of nitrogen atoms into the carboxylic ring [26]. For instance, prawn shells have been used to fabricate NCQDs with an average diameter of 4 nm and 9% quantum yield [65]. The NCQDs solution (20 µg/mL) exhibited yellowish and blue luminescence under normal light and UV excitation wavelengths of 365 nm as well as a maximum emission intensity at 405 nm under excitation at 330 nm. The fluorescence response of these NCQDs to Cu^2+^ ions has been evaluated. NCQDs exhibited good selectivity toward Cu^2+^ (with a detection limit of 5 nm) in the presence of various metal ions, including Co^2+^, Hg^2+^, and Fe^2+^.

Jiang et al. (2020) demonstrated the capability of NCQDs to be considered fluorescent sensors. NCQDs (with a diameter of approximately 4.21 nm) were obtained from commercial chitin via a hydrothermal process and were used for detecting hypochlorite ions (ClO^−^) [26]. These NCQDs displayed strong fluorescence with a quantum yield of 25.8%. The authors attributed this high quantum yield to ammonia, an additional nitrogen source in the synthesis process. The optical properties of such NCQDs under daylight and a UV lamp of 365 nm exhibited a light-yellow color and brilliant blue fluorescence, respectively. These NCQDs have blue fluorescence with a maximum emission intensity at 433 nm under excitation at 360 nm. The detection limit of ClO^−^ was 1.47 µm in the presence of different ions and anions, such as Ca^2+^, Co^2+^, Fe^2+^, I^−^, Cl^−^, and HCO^3−^.

Chitin-based materials have also been used as precursors for the preparation of NCQDs. Chitin nanofibers (CNFs) were used as a new carbon source to obtain NCQDs via a microwave-assisted hydrothermal route [66]. The NCQDs were synthesized with a spherical shape, a size of about 2–12 nm, and a quantum yield of 5.1%. The optical properties of the NCQDs revealed strong blue fluorescence with a maximum emission at 480 nm under excitation at 370 nm. In addition, the fluorescence of the as-prepared NCQDs was quenched with Cu2+ ions. The results showed that the NCQDs could be a sensing system for determining D-penicillamine (DPA). A good selectivity toward DPA was found in the presence of several molecules, such as ascorbic acid, tyrosine, cysteine, glycine, uric acid, and even ion metals (Ca^2+^, K^+^, Mg^2+^, and Cl^−^).

#### 3.2.2. Chitosan

Chitosan is a partially deacetylated form of chitin. In chitosan, a fraction of the N-acetyl-D-glucosamine is converted into D-glucosamine. Chitosan is a biopolymer featuring excellent properties such as biocompatibility, biodegradability, and non-toxicity [91,92,93]. Chitosan has been used as a precursor for the synthesis of CQDs. For instance, Feng et al. (2022) synthesized CQDs using chitosan as a precursor following a hydrothermal carbonization route [27]. They obtained CQDs with 4–11 nm diameters and a quantum yield of 16.81%. Other researchers have reported chitosan-derived CQDs with higher quantum yield values. Quantum yield values of 31.8% have been reported using a hydrothermal carbonization route [72] and 27% through microwave-assisted hydrothermal carbonization [43].

The optical properties of chitosan-derived CQDs have been assessed. These CQDs have been excited at different wavelengths to study their fluorescent behavior. For a maximum excitation peak at 510 nm, a maximum emission peak was found at 550 nm [73]. Wang et al. (2016) evaluated the fluorescence response of CQDs to detect Hg^2+^ [72]. These CQDs showed excellent selectivity toward Hg^2+^ in the presence of Co^2+^. Other authors have studied the detection of different metal ions, such as Fe^3+^, Ag^+^, Cr (IV), and NO^2-^ [27,74], demonstrating good selectivity and sensitivity of these chitosan-derived CQDs in the presence of metal ions.

Mu et al. (2021) employed chitosan-derived CQDs synthesized via hydrothermal carbonization to detect alkaline phosphatase (ALP) [24]. These CQDs were 1.5–3.1 nm in diameter with an amorphous structure. The optical properties of such CQDs exhibit strong blue fluorescence with a maximum emission at 412 nm under an excitation wavelength of 290 nm and with a fluorescence lifetime of 5.56 ns. These CQDs were used to develop a novel ratiometric fluorescence assay. The fluorescence was quenched by adding Eu^3+^ and calcein. This system (CQDs + calcein + Eu^3+^) was used for the detection of alkaline phosphatase. In the presence of alkaline phosphatase, calcein significantly increased its fluorescence intensity. In contrast, the fluorescence of CQDs decreased as the concentration of alkaline phosphatase in the assay increased. The assay showed excellent selectivity for ALP detection in the presence of different enzymes (HRP, TG, ATP, and β-GC).

### 3.3. Polysaccharides Extracted from Fish and Other Marine Animals

Fish scales are plywood-like structures of closely packed collagen fiber layers reinforced with a mineral phase of calcium-deficient hydroxyapatite [96,97]. Alongside collagen, chitin and gelatin are also components of interest in fish scales. These components make the scales rich not only in carbon and hydrogen but also in oxygen and nitrogen [88]. Zhang et al. [98] prepared highly fluorescent carbon dots with the incorporation of N and O functionalities through a hydrothermal reaction using fish scales of the crucian carp as a precursor. These CQDs exhibited strong fluorescent emissions at 430 nm, with a relative quantum yield of 6.9%, low cytotoxicity, and robust fluorescence stability against photobleaching. They found that these CQDs can be quenched by Fe^3+^ ions, which enables their application as fluorescent Fe^3+^ nanoprobes. Wu et al. [99] used fish-scale wastes for the preparation of N-doped photoluminescent QCDs with a nitrogen content of 14.6%. The prepared CQDs were 2 nm in size and displayed a narrow photoluminescence emission band (400–490 nm), with a high quantum yield up to 17%.

There are other polysaccharides that can be extracted from other marine animals. For instance, chondroitin sulfate is a long, linear polysaccharide extracted from cartilaginous marine animals such as sharks, sturgeons, and squid. Kim et al. (2020) synthesized CQDs via hydrothermal carbonization from chondroitin sulfate polysaccharide obtained from shark cartilage [41]. The fabricated CQDs, with diameters ranging from 19 to 60 nm and an intrinsic quantum yield of 20.46%, exhibited multicolor photoluminescent properties (Table 2). They showed a clear yellowish-brown color under daylight as well as a yellowish-green and light blue under UV excitation at 430–440 nm and 365 nm, respectively. In addition, they exhibited a maximum emission intensity at 490 nm under 390 nm excitation. Due to their excellent photoluminescence properties and low toxicity, they were used for in vivo bioimaging of zebrafish larvae. They emitted green and blue fluorescence emissions in the gut of such larvae. 

## 4. Processing Routes of Marine Polysaccharide-Based CQDs

A variety of processing routes has been developed to synthesize CQDs. The processing routes most commonly used with marine polysaccharide precursors include hydrothermal carbonization, microwave-assisted-hydrothermal synthesis, thermal acid dehydration, solvothermal dehydration, and pyrolysis. Table 2 shows the different processing routes reported for synthesizing CQDs from marine polysaccharides.

### 4.1. Hydrothermal Carbonization

**Figure 6:**
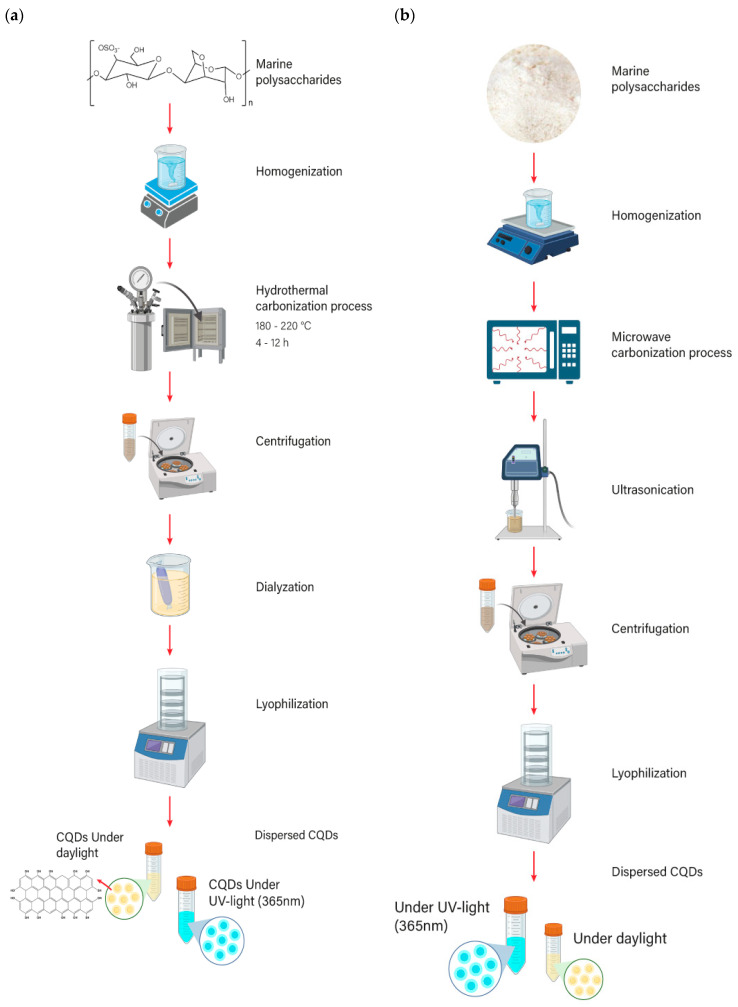
Processing routes used to produce CQDs by (**a**) using hydrothermal carbonization and (**b**) microwave.

Hydrothermal carbonization is the most common processing route used to synthesize single- and doped-carbon quantum dots. The parameters used in the carbonization reaction (i.e., temperature, pressure, and time) and the carbon source influence the final properties of CQDs, such as size, quantum yield, and optical properties [23]. It features a facile one-step synthesis procedure, low cost, and is referred to as an environmentally friendly process [22]. Following this route, quantum dots are synthesized from the homogenization of carbon-rich precursors (i.e., marine polysaccharides) in an aqueous solution in a pressure vessel (Figure 6a). After the carbonization is finished, the carbon dots are present as an aqueous sludge. Then, waste products and excess fluid need to be removed using complementary procedures such as dialysis and freeze drying, among others, to obtain a solid powder of carbon quantum dots. Even many authors have reported the use of syringe filter membranes (0.22 µm–0.45 µm) at different stages of the carbonization process to obtain transparent solutions before pouring them into an autoclave reactor and to remove the carbonized precipitate (Figure 6a).

Overall, the mechanism during hydrothermal carbonization to obtain CQDs includes depolymerization, dehydration, carbonization, aromatization, nucleation, and growth [52,53,72]. First, marine polysaccharides are depolymerized or fragmented, and continuous intramolecular dehydration occurs during the heating process. Re-polymerization and aromatic clusters (C=C double bonds and aromatized molecules) are formed from the molecules obtained under carbonization conditions. Nucleation and growth of carbon substances will occur instantaneously after the concentration of aromatic clusters in the aqueous solution reaches the critical supersaturation point [52,53,72]. 

Previous steps are sometimes needed before proceeding with hydrothermal carbonization. Sun et al. (2021) synthesized nitrogen-doped carbon quantum dots from chitosan [67]. They reported that prior to carbonization, the chitosan mixture was filtered through a 0.45 µm membrane to remove any insoluble substances. The purified mixture was poured into an autoclave at 180 °C for 12 h. The carbon dots obtained after the process were subjected to a second filtration through a 0.22 µm membrane and finally centrifuged for 15 min. Supchocksoonthorn et al. (2021) reported the use of hydrogen peroxide as a catalyst, which was used to form hydroxyl radicals that can react with the carbon precursors (κ-Carrageenan) and generate hydrophilic functional groups on the resulting CQDs [55]. In order to neutralize the as-prepared CQDs, NaOH was used. The same procedure was used by Zhou et al. (2016) to develop CQDs from alginate [22]. The authors used ultrasonication to homogenize sodium alginate with hydrogen peroxide at 3%. The homogenized mixture was then transferred into a sealed autoclave where carbon dots were synthesized. The temperature used by the autoclave was in the range of 180 °C–240 °C for 12–24 h. The carbon dots produced were filtered through a 0.22–0.45 µm nylon membrane in order to remove the unreacted or non-solubilized parts [22].

### 4.2. Microwave-assisted-hydrothermal Synthesis

Another facile and low-cost procedure to obtain CQDs is the microwave-assisted method (Figure 6b). Compared to hydrothermal carbonization, the advantage of using this method is that a shorter carbonization reaction period is needed. Other advantages include uniform heating, less power consumption, and the size-controllable dots that can be obtained [23]. This microwave-assisted method has been used to synthesize CQDs from k-carrageenan [57], sodium alginate [90] and chitosan [86]. For instance, Xiao et al. (2013) synthesized CQDs from chitosan [86]. An aqueous chitosan solution was first transferred to a microwave oven, where solvent molecules provided uniform heating, leading to the formation of CQDs. This procedure was carried out over minutes. After the sample was cooled down to room temperature, it was diluted in distilled or double-distilled water while being stirred and sonicated for 10–30 min. Then, the sample was centrifuged at a high rotation speed for 15–30 min to obtain a solution of CQDs. Finally, the sample was freeze-dried to obtain a powder of CQDs. 

### 4.3. Other Processing Routes

In addition to hydrothermal and microwave processes, pyrolysis has been performed in argon atmosphere at 900 °C to obtain multilayer graphitic quantum dots derived from alginate. This multilayered graphitic structure can be converted into graphene quantum dots after laser ablation treatment. As a result, the dimensions of the alginate-derived graphene quantum dots vary as a function of the number of laser pulses and the ablation time [59].

Chauhan et al. [40,61,89] reported using an incineration process (450 °C–600 °C) to prepare CQDs from agarose gel waste. After cooling down to room temperature, a blackish-brown powder was obtained. The powder obtained was dispersed in deionized water and centrifuged. The collected supernatant was sieved through a 0.22 µm filter membrane and heated at 100 °C–130 °C for 1–2 h to obtain powder CQDs.

An ultrasonic-assisted nanoprecipitation in acidic solvent can also be used to synthesize CQDs [60]. It involves the formation of polysaccharide nanoparticles using an ultrasonic approach with a strong acid solvent as a dehydrating agent. Then, CQDs are prepared by carbonization of the as-prepared nanoparticles.

The solvothermal dehydration method is a variation of the hydrothermal method in which ammonia, alcohol, or an inorganic solvent is used instead of water [103]. Zhang et al. [81] utilized chitosan as a precursor to synthesize carbon dots through solvothermal carbonization. In this case, ethanol was used as the solvent, and the resulting product was suggested to have utility in electrochemical imaging sensors.

Zattar et al. [75] utilized a thermal acid dehydration technique to synthesize carbon quantum dots (CQDs) from chitosan. In the first stage, a carbonaceous material was obtained after the combination of sulfuric acid and chitosan at 80° C under magnetic stirring at 700 RPM for 40 min. The reaction was stopped by adding distilled water. Water and ethanol were subsequently used to wash the product by vacuum filtration to remove the excess of acid. The material was then dried in an oven at 70 °C, followed by oxidation through the addition of nitric acid. Carbon dots were finally obtained after undergoing a second heating process, neutralization, and centrifugation of the material.

Based on the above, one of the parameters used to evaluate the convenience of using a specific processing route is the synthetic yield. Synthetic yield (SY), also known as product yield (PY), is defined as the efficiency of converting the precursor into CQDs. It is calculated as the ratio between the mass of the obtained CQDs (m_CQDs_) and the precursor’s mass (m_precursor_). SY is calculated following Equation (2).

(2) $${SY}_{CQDs}=\left[\frac{m_{CQDs}}{m_{precursor}}\right]\times 100\%$$

Table 1 shows the yield (SY) for several processing routes used to synthesize CQDs from marine polysaccharides. The highest SY (90%) was obtained through hydrothermal carbonization using chitosan as a precursor to synthesize solid-state carbon dots (SS-CDs).

## 5. Applications

### 5.1. Drug Delivery

**Figure 7:**
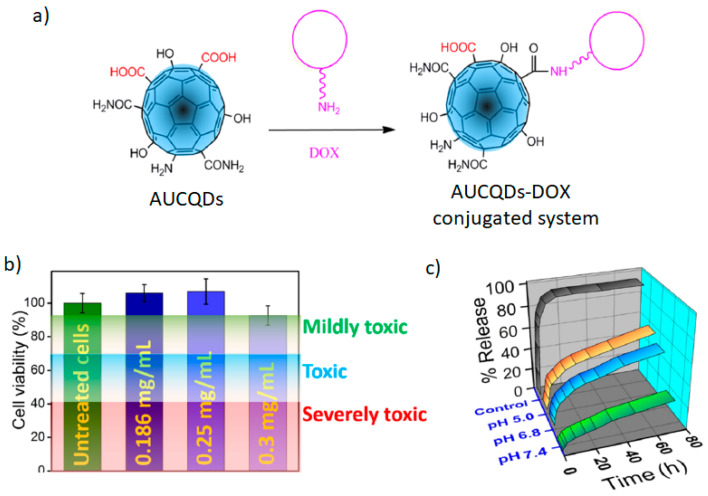
Schematic presentation (**a**) for the synthesis of doxorubicin (DOX) onto the surface of alginate-urea CQDs (AUCQDs–DOX); (**b**) in vitro toxicological assessment of AUCQDs in three different concentrations; (**c**) cumulative release of DOX from the AUCQDs–DOX conjugated systems at different pH values. Reproduced with permissions from [90].

Fluorescent CQDs based on sodium alginate and urea (AUCQDs) were used to load a chemotherapeutic agent known as DOX (doxorubicin). The strategy of using sodium alginate and urea was to obtain different functional groups (–OH, –COOH, and –CONH_2_) attached to the surface of the alginate-urea CQDs that promote interactions with drug molecules [90] (Figure 7a). As a result, the AUCQDs at concentrations of 0.186–0.3 mg/mL were not toxic to MCF-7 human breast cancer cell lines (Figure 7b). The loading efficiency of DOX onto the surface of AUCQDs was 70% at pH 7.4. The cumulative release of DOX at different pH values was studied over 80 h (Figure 7c). It was found that around 20% of the release takes place after 72 h. This gradual release is associated with better control of DOX administration, thus preventing the initial-burst drug release when no CQDs carrier is used (95% after 20 min).

In another study, CQDs based on κ-carrageenan and phenyl boronic acid were used to load metformin, an anti-diabetic drug for diabetes mellitus. The drug-loading efficiency was 71.6%, and the drug release was around 60% during the first ten minutes [54]. In comparison with the accumulative release of pristine drug, which showed a burst-release phenomenon (92% after 5 min), the CQDs based on κ-carrageenan showed potential for use as a vehicle for metformin delivery.

In addition to drug delivery, CQDs derived from marine polysaccharides can be employed for gene delivery. Currently, no gene vector possesses the property of self-imaging. In this context, sodium-alginate-derived CQDs were proposed to act as a self-tracking gene carrier [22]. The complexes (CQDs/pDNA) were obtained using plasmid TGF-b1 (pDNA) and the alginate-derived CQDs. The results showed that the CQDs/pDNA were not cytotoxic against 3T6 cell lines. Furthermore, the CQDs/pDNA complex produced a transfection efficiency similar to Lipofectamine 2000, a common transfection reagent. The process of entering cells was monitored using green and blue fluorescence. The blue fluorescence corresponds to the CQDs/pDNA complex and allows it to be located at the periphery of the nucleus, while the green fluorescence derived from YOYO-1 dye contributes to the visualization of pDNA transfection in the nucleus. These results indicated that the alginate-derived CQDs are nanomaterials with the potential for dual roles as both gene carriers and bioimaging probes.

### 5.2. Bioimaging

Due to carbon dots’ fluorescence properties, many researchers aim to develop in vitro and in vivo monitoring and detection applications to diagnose cancer cells and other diseases. In this regard, developing probes for detecting abnormal levels of alkaline phosphatase, a biomarker for detecting diseases and cancer cells in human serum, is vital. For this reason, Mu X. et al. (2021) developed a fluorescent probe from chitosan-doped carbon dots using chitosan as a precursor with calcein to detect alkaline phosphatase [24]. To detect alkaline phosphatase in cells, the researchers evaluated the cell viability of carbon dots using HepG2 cells. They obtained more than 80% viability at concentrations greater than 0.05 mg/mL of carbon dots. Thanks to their biocompatibility and selectivity for alkaline phosphatase detection, they evaluated the intracellular bioimaging of alkaline phosphatase in HepG2 cells. HepG2 cells incubated with the carbon dots and calcein mixture showed strong green fluorescence from calcein and weak blue fluorescence from carbon dots. However, cells incubated with the mixture of carbon dots, calcein, and Eu^3+^ showed a decrease in green fluorescence from calcein, as determined in their previous fluorescence studies. When pNPP was added to the incubation medium, it was hydrolyzed by endogenous alkaline phosphatase, generating PO_4_^3−^. This phosphate anion combined with Eu^3+^, which released free calcein. Consequently, it caused an increase in the green fluorescence intensity of calcein in HepG2 cells and a decrease, an almost complete quenching, of the blue fluorescence intensity of the carbon dots.

**Figure 8:**
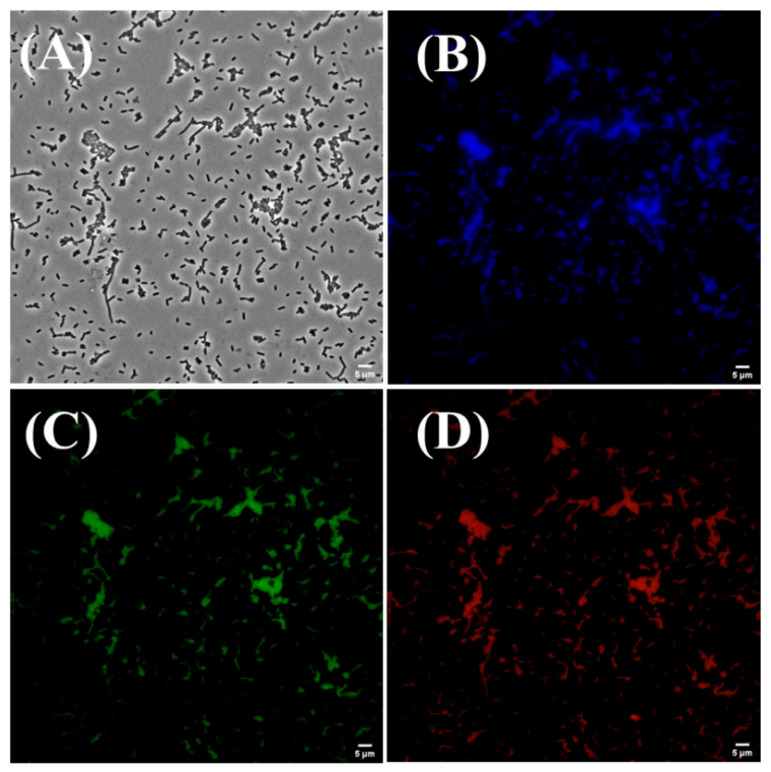
Fluorescence microscopic images of *E. coli* incubated with nitrogen-doped carbon dots under (**A**) bright field, scale bar: 5 µm; (**B**) 405 nm, scale bar: 5 µm; (**C**) 470 nm, scale bar: 5 µm; (**D**) 560 nm, scale bar: 5 µm. Reproduced with permissions from [67].

On the other hand, Sun et al. (2021) reported on the bioimaging of *E. coli* and *B. subtilis* bacteria using a fluorescent probe made from nitrogen-doped carbon dots [67]. For this reason, they performed toxicity studies of the carbon dots derived from chitosan at different concentrations (20–400 µg/mL) for the two bacteria used in the research. The results showed more than 93% bacterial cell viability, indicating low cytotoxicity of the nitrogen-doped carbon dots. The bacteria incubated with the carbon dots showed blue, green, and red fluorescence at different excitation wavelengths: 405 nm, 470 nm, and 560 nm, respectively (Figure 8). The results in the fluorescence images indicated that the carbon dots could pass through the cell membranes, causing bacterial cell staining. 

### 5.3. Biosensing

Due to their luminescence properties, fluorescent stability, and good biocompatibility, carbon dots present a promising perspective as fluorescent carbon nanomaterials for molecular biological sensing. According to the literature, a high level of iron ion content and/or iron deficiency in the body could increase human health problems. For this reason, it is essential to develop effective and straightforward methods for detecting ionic iron. In this instance, Liu et al. (2019) developed a carbon probe for the selective and sensitive detection of Fe^2+^ ferrous ions using carbon dots derived from chitosan and acrylamide [46]. To perform the arrest, they analyzed the fluorescence intensities of the carbon dots, which showed chelating metal properties in the presence of different metal ions, including Pb^2+^, Cr^3+^, Cu^2+^, Hg^2+^, Fe^3+^, and Fe^2+^, among others, at a concentration of 300 µm. The fluorescence intensity of the carbon dots in the presence of Fe^2+^ was quenched, with a 55% attenuation. Xu et al. (2021) reported the sequential detection of Fe^3+^ iron ions and ascorbic acid through probes based on carbon dots derived from chitosan and κ-carrageenan co-doped with nitrogen and sulfur [100].

**Figure 9:**
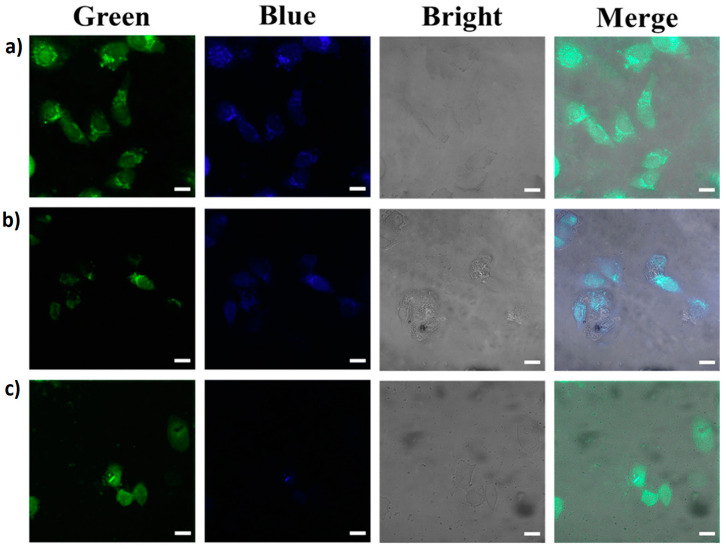
Fluorescence microscopic images of HepG2 cells incubated with chitosan-derived CQDs and (**a**) calcein, scale bar: 20 µm; (**b**) calcein and Eu^3+^, scale bar: 20 µm; and (**c**) calcein, Eu^3+^ and *p*-nitrophenyl phosphate, scale bar: 20 µm. Reproduced with permissions from [24].

Mu et al. (2021) developed a fluorescent probe with good stability based on the ratiometric fluorescence method from chitosan-doped carbon dots, using chitosan and ethylenediamine as precursors together with the metal ion indicator calcein, for the detection of alkaline phosphatase [24]. Different assays were performed to detect alkaline phosphatase by measuring ratiometric fluorescence through fluorescence emission spectra. In the first instance, they demonstrated the quenching effect that calcein has on the carbon dots, presenting two fluorescence emission peaks. For the case of the carbon dots, the peak was less than 1000 a.u. at about 412 nm emission wavelength, and for calcein, the peak was 4000 a.u. at about 512 nm emission wavelength. The fluorescence signal of calcein was quenched when Eu^3+^ was added to the mixture. However, by adding the enzyme alkaline phosphatase at different concentrations in the range of 0.09–2 mU/mL to the mixture of carbon dots, calcein, and Eu^3+^, they saw an increase in the fluorescence intensity of calcein and a decrease in the fluorescence intensity of carbon dots (Figure 9). Subsequently, in order to evaluate the ratiometric fluorescent detection of alkaline phosphatase activity, they used human serum samples. They determined that their detection was 0.295 to 0.297 mU/mL of alkaline phosphatase in 100-fold diluted human serum.

A non-enzyme-based glucose sensor for determining blood sugar using boronic-acid-functionalized κ-carrageenan CQDs was developed [54]. The authors developed a glucose sensor utilizing a Whatman paper strip soaked in a CQDs dispersion. The paper strip was then dried. The biosensor based on κ-carrageenan CQDs features linearity in the range of 0–210 µM, with a detection limit at a concentration of 1.7 µM. The fluorescence quenching revealed that the paper-based sensor was selective for glucose over other molecules such as fructose, lactose, and maltose. Another important biomolecule is dopamine, which is essential to monitor in order to diagnose mental diseases such as Parkinson’s, schizophrenia, and bipolar disorder, among others. Chauhan et al. (2021) reported a noticeable fluorescence quenching of agarose waste CQDs toward dopamine, with a detection limit of 0.128 µM [61]. In addition, they proved the selectivity of dopamine over more than ten interferences, including biomolecules (glycine, glucose, ascorbic acid, and urea), amino acids (ι-Serine, ι-Valine, ι-cysteine, and ι-alanine), and ions (Na^+^, K^+^, Mg^2+^, and Ca^2+^). 

Das et al. (2018) developed a fluorescence sensor using nitrogen- and sulfur-co-doped CQDs to monitor the acetone level in biological samples such as blood and urine [53]. These co-doped CQDs (NSCQDs) were synthesized using urea and κ-carrageenan. The NSCQDs revealed effective selectivity for acetone molecules in biological fluids, with a detection limit of 7.2 × 10^−7^ M. The fluorescence properties showed that acetone could quench the NSCQDs in a range of 0–0.05 M and 0–0.01 M for blood and urine samples.

### 5.4. Sensing

It is important to assess water quality according to the ion and metal content as well as environmentally polluting dyes. For example, Jun Zhan et al. (2019) used carbon dots to detect metal ions [78]. The fluorescence-quenching tests with different metal ions determined that CQDs exhibited higher detection selectivity for Fe^3+^ at 1 mM. They also studied the carbon dots’ quenching behavior at different Fe^3+^ concentrations. As a result, they found that as the concentration increased, the fluorescence intensity decreased. The quenching of the fluorescence of the carbon dots in the presence of the metal ion Fe^3+^ was due to chelation between the ion and the phenolic hydroxyl groups.

Fong et al. (2015) synthesized alginate CQDs and used them to evaluate their fluorescence properties for sensing ferric ions [60]. CQDs fluorescence was quenched at different concentrations of Fe^3+^ (0–25 µM). The result exhibited good selectivity toward ferric ions (Fe^3+^) in the presence of several cations, including Na^+^, Mg^2+^, Cr^2+^, Mn^2+^, Co^2+^, Cu^2+^, Hg^2+^, and Pb^2+^, with a detection limit of 1.06 µM. 

Wang et al. (2021) used CQDs derived from κ-carrageenan as a fluorescent detector of metal ions [49]. Such CQDs exhibited high fluorescence intensity and excellent stability, with a quantum yield of 20.6%. The fluorescence intensities of these CQDs were modified with the addition of Fe^3+^ and oxytetracycline (Otc), demonstrating their good selectivity and sensitivity for the detection of metal ions. The detection limit was 0.21 µM in the presence of ions such as Al^3+^, Ca^2+^, Pb^2+^, Na^2+^, and Hg^2+^, among others. With the addition of Otc, these CQDs showed a detection limit of 0.05 µM in the presence of ions such as Na^+^, Mg^2+^, Ca^2+^, Zn^2+^, and SO_4_^−^ as well as other substances such as sucrose, glucose, glycine, urea, ethanol, vitamin C, and oxalic acid.

Sun et al. (2021) reported the synthesis of carbon dots from N-doped chitosan to detect nitrite (NO^2−^) present in tap water and lakes [67]. On the other hand, Sun et al. (2020) reported carbon dots synthesized from carbon alginate with good fluorescence properties in mild acidic/basic and metal ion environments [58]. Bera and Mohapatra (2020) developed a sensor/probe based on chitosan-derived carbon dots integrated with CdTe to detect organophosphate herbicides such as glyphosate in an aqueous medium [77]. The detection was based on the photoelectron transfer strategy. The fluorescence of CdTe in the presence of glyphosate turns on as the disintegration of CdTe and carbon dots occurs.

Chauhan et al. (2020) reported the use of CQDs derived from agarose gel waste to develop a fluorometric sensor for detecting Zn^2+^ and CO_3_^2−^ ions [40]. The data showed that the detection limit of the agarose-waste CQDs sensor was 0.26 nM and 0.17 nM for Zn^2+^ and Co_3_^2−^ ions, respectively. These lower concentration levels can provide green, non-toxic, and cost-effective solutions for water treatment. 

Several industries, including textiles, cosmetics, and paints, use large quantities of synthetic dyes that harm aquatic ecosystems. Recently, Leuterio et al. (2022) compared the degradation of methylene orange dye using (к-, ɩ-, and λ-) carrageenan CQDs and galactose CQDs [42]. The authors proved that the sulfate content in carrageenan CQDs plays a critical role in the degradation of the methylene orange dye. It is related to the acidic nature of CQDs based on carrageenan that allows the dye to be protonated, thus destroying its aromatic structure and degrading it over time. The dye degradation efficiency was 3%, 21%, and 22% using к-, λ-, and ɩ-carrageenan CQDs. Compared to CQDs based on galactose (Gal-CQDs) with the same sugar structure as carrageenan but with no ester sulfate groups, the results showed that Gal-CQDs did not affect the methylene orange dye, and no degradation efficiency was reported. 

### 5.5. Food

Recently, interest in applying carbon dots in the food industry has increased. Carbon quantum dots derived from polysaccharides exhibit antioxidant and antibacterial activities, excellent biocompatibility, and non-toxicity. Additionally, they can provide UV protection. Based on the above, CQDs can be used as additives for manufacturing packaging materials, as sensors to evaluate food quality and safety, and even as a tool to study plant-related diseases [57,104].

For instance, Fu et al. (2022) developed an improved bio-nanocomposite film by integrating chitosan-derived carbon dots through hydrothermal synthesis into the mixture of gelatin and chitosan [31]. Photoluminescence studies of the carbon dots showed that they could emit blue light with a peak wavelength of 420 nm. The composite film of gelatin/chitosan/carbon dots presented a homogeneous and flexible appearance. When exposed to sunlight, the films became more yellowish as the content of carbon dots (0–20%) in their composition increased. Moreover, the fluorescence of the films increased when exposed to UV light. This fluorescence property of the carbon dots was used as an indicator of the fish’s freshness since changing the fish’s pH changes the film’s fluorescence brightness. On the other hand, antibacterial activity studies of the film were carried out using *E. coli* (Gram-negative) and *S. aereus* (Gram-positive) bacteria. It showed significant increases in the zone of inhibition in films composed of carbon dots.

Organic compounds such as capsaicin are an essential indicator of the quality of commercial spicy foods. The study of ref. [55] (2021) evaluated capsaicin detection using ɩ-carrageenan CQDs. The authors found a detection limit of 5.4 nM, a linear range from 0.05–500 µM, and good selectivity towards capsaicin over other molecules found in food, including glucose, citric acid, and ascorbic acid. 

Sun et al. (2020) reported the use of carbon dots derived from sodium alginate and glutaraldehyde through hydrothermal carbonization [58]. These were added to sodium-alginate films to prevent their aging when exposed to UV radiation. The films were uniform in color and transparent. Since these films have applications for food coating, a cell viability test was performed on them, indicating that the carbon dots showed more than 72% cell viability at different concentrations (0.16, 0.32, 0.61, 1.25, and 2.50). Additionally, the transmittance of the films with carbon dot additives was 4.8% in the UV region, a value much lower than the film without additives, which was 95.1%. Tensile strength tests were carried out to verify their anti-ultraviolet performance. The films were subjected to UV irradiation for three days, and when tested, it was confirmed that they only showed a 15% decrease in the initial resistance. This property was attributed to the fact that the carbon dots converted the absorbed UV light into thermal energy, accelerating the loss of water molecules and making the internal structure of the films more compact. These results revealed that carbon dots as additives provided the property of UV-initiated ageing resistance to sodium-alginate films.

## 6. Future Perspective

Polysaccharides are the most abundant and complex organic molecules in the ocean [38]. A wide range of CQDs can be obtained by utilizing a diverse selection of polysaccharides as both precursors and doping agents. The main challenges that hinder the full development of a novel carbon dots industry are related to the processing routes and the photoluminescence characteristics of CQDs. Regarding processing methods, the main challenge is still to produce CQDs with high QYs using simple, one-step techniques. Novel methodologies must enable the control of optical properties to achieve tunable emission. Most investigations on CQDs have shown that it is relatively easy to synthesize blue- and green-fluorescent carbon nanoparticles with high fluorescence quantum yields [105]. In fact, CQDs typically exhibit strong absorption in the ultraviolet (UV) range and dominant emissions in the blue range [106]. However, for applications in the biological field, red-luminescent CQDs are needed because blue and green emissions cannot penetrate tissue deeply and are likely to cause auto-fluorescence in biosamples [107,108]. Therefore, the synthesis of CQDs with strong red/near-infrared (NIR) emission/excitation has been identified as a crucial factor in advancing their applications in the biomedical field [109,110,111].

Zhu et al. [106] reviewed the process of producing red CQDs from various precursors. They discovered that several of the precursors utilized in synthesizing red CQDs contain heteroatoms that create new energy bandgaps, resulting in a shift towards red-shifted spectra. Marine polysaccharides such as carrageenan and chitosan feature sulfur and nitrogen heteroatoms, which could be used to fabricate red CQDs with narrowed electronic bandgaps. Future studies should develop processing routes that capitalize on the heteroatoms found in marine polysaccharides to produce red-emitting CQDs.

## 7. Conclusions

In this review, we report that marine polysaccharides extracted from sources such as algae, crustacean shells, and fish waste are excellent precursors for obtaining carbon quantum dots (CQDs). These polysaccharides contain heteroatoms such as nitrogen, sulfur, and oxygen in their structure, which makes them ideal for CQD synthesis. CQDs doped with these heteroatoms show improvements in their optical properties, photoluminescence, quantum yield, and quenching mechanism. These essential properties make CQDs applicable in several fields, such as drug delivery, bio-imaging, bio-sensing, and metal ion detection. Further studies will enable the development of new types of CQDs, including red-emitting CQDs, which will expand the range of applications for CQDs in the biomedical field.

This review will guide researchers to explore new sources such as marine microorganisms or microalgae biomass, which can be used as precursors for carbon quantum dots, generating a new area of research.

## Figures and Tables

**Table 1:** Marine polysaccharides classified by origin and main properties of CQDs synthesized using them as precursors.

Source	Marine Polysaccharide	CQDs Properties					
		Size (nm)	Color	Synthetic Yield (%)	Quantum Yield (%)	Heteroatoms Content (%)	Ref.
Marine algae	Carrageenans	1.5–30 0.63–3.92 ^c^	Yellow-brown ^a^, Yellow ^a^, Green ^b^	4.4	2–69.27	S%: 2.7–5.29% N%: 8.18%	[42,52,53,54,55,56,57]
	Alginates	2–20 100	Yellow ^a^, Brown ^a^, Green ^b^, Blue ^b^	–	5.42–48.7	N%: 0.02%	[22,58,59,60]
	Others	1–40	Dark brown ^a^ Green ^b^, Blue ^b^	–	56–62	O%: 26.98–32.34% S%: 3.15% N%: 2.41–8.12%	[40,61,62,63,64]
Crustaceans	Chitins	1–20.5	Yellow ^a^, Brown ^a^, Orange ^a^, Blue ^b^, Green ^b^	6.7–9.9	5.1–35	O%: 4.67–28.54% S%: 0.38–1.54% N%: 2.63–18.25%	[26,65,66,67,68,69,70,71]
	Chitosans	0.6–11 2.7–3 ^c^	Transparent ^a^, Brown ^a^, Yellow ^a^, Blue ^b^, Green ^b^	85.3–90	3.3–40	O%: 1.47–51.67% N%: 0.02–9.63%	[24,27,29,31,72,73,74,75,76,77,78,79,80,81,82,83,84,85,86,87]
Fish	Chondroitin sulfate (shark cartilage)	19.6–60	Yellow-brown ^a^ Yellowish-green ^b^ Blue ^b^	–	20.46	C% and O% > 90% S% < 10%	[41]
	Collagen and chitin (Fish scales)	13	–	–	17.3	O%: 26% N%: 8%	[88]

^a^, under daylight; ^b^, under UV lamp; ^c^, hydrodynamic size obtained by dynamic light scattering.

**Table 2:** Processing routes, properties, and applications of carbon quantum dots derived from marine polysaccharide.

Precursor Marine Polysaccharide	Processing Route	Size (nm)	Color	Quantum Yield (%)	Maximum Excitation Wavelength (nm)	Maximum FL Emission Wavelength (nm)	Applications	Ref.
***Algae***								
κ-Carrageenan	Hydrothermal carbonization	0.93–2.47 ^c^	Yellow-brown ^a^ Green ^b^,	–	340	435	Degradation of dyes	[42]
κ-Carrageenan	Hydrothermal carbonization	2.1	Yellow ^a^	–	340	454	Antitumor, antiviral	[52]
κ-Carrageenan	Hydrothermal carbonization	1.5–5.5	Yellow ^a^, Green ^b^	69.27	360	432	Acetone sensing	[53]
κ-Carrageenan	Hydrothermal carbonization	2.75–6.25	Green ^b^	62.54	340	448	Cr (VI) in environmental water and intracellular imaging	[56]
κ-Carrageenan	Hydrothermal carbonization	3.2	Yellow ^a^, Bluish green ^b^	14.64	380	470	Drug Delivery of anti-diabetic drug Metformin	[54]
κ-Carrageenan	Hydrothermal carbonization	1.8	Yellow ^a^, Green ^b^	20.6	340	420	Sensing of Fe^3+^	[49]
κ-Carrageenan	Microwave	25–30	Brownish-yellow ^a^, Green ^b^	21	340	434	Bioimaging of plant cell biology	[57]
ɩ-Carrageenan	Hydrothermal carbonization	2–6	Yellow ^a^	2	360	472	Capsaicin sensing	[55]
ɩ-Carrageenan	Hydrothermal carbonization	0.68–3.92 ^c^	Yellow-brown ^a^, Green ^b^	–	340	435	Degradation of dyes	[42]
λ-Carrageenan	Hydrothermal carbonization	0.63–1.37 ^c^	Yellow-brown ^a^, Green ^b^	–	320	425	Degradation of dyes	[42]
Porphyra polysaccharide	Hydrothermal carbonization	1–9	Blue ^b^	56.3	360	~450	Bioimaging	[63]
Sodium alginate	Hydrothermal carbonization	5–10	Blue ^b^	12.7	300	450	Gene delivery and bioimaging	[22]
Sodium alginate	Hydrothermal carbonization	2–5	Brown ^a^, Blue ^b^	11	360	467	Anti-ultraviolet ageing additives	[58]
Sodium alginate	Microwave-irradiated thermal coupling method	5.6	Yellow ^a^, Green ^b^	48.7	330	~405	Drug delivery	[90]
Sodium alginate	Ultrasonic-assisted nanoprecipitation in acidic solvent	100	–	5.42	340	440 nm	Fe^3+^ sensing	[60]
Sodium alginate	Laser ablation–pyrolysis	10–20 nm (length) 1–1.5 nm (depth)		–	350	430	–	[59]
Fucoidan	Hydrothermal carbonization	4–10	Dark brown ^a,^ Green ^b^	–	362	453	Endodontic infections	[62]
Agarose	Hydrothermal carbonization	8–10	Black-brownish ^a,^ Blue ^b^	56	310	550	Dopamine sensing	[61]
Agarose	Thermal treatment	2–10	Milky coloration ^a^, Blue ^b^	62	300	~420	ʟ-Aspartic acid sensing	[40]
Ulvan	Hydrothermal carbonization	20–40	–	0.68	360	435	Detection of nonylphenol	[64]
***Crustacean***								
Chitin	Hydrothermal carbonization	4	Yellow ^a^, Blue ^b^	9	330	405	Cu ^2+^ sensing	[65]
Chitin (DA ≥ 90%)	Hydrothermal carbonization	4.21	Yellow ^a^, Blue ^b^	25.8	360	433	ClO ^−^ sensing	[26]
Chitin (85% DD)	Hydrothermal carbonization	2.8	Yellow ^a^, Blue ^b^	35	330	400	Bacterial imaging	[67]
Chitin	Hydrothermal carbonization	1–10	Yellow ^a^, Blue ^b^	5.77	380	~470	Fe^3+^ sensing	[68]
Chitin	Hydrothermal carbonization	4–8	Brown ^a^, Blue ^b^	–	300	440	–	[69]
Chitin	Ionic liquid + thermal treatment	2.8	Orange ^a^	–	400	503	Non-Newton nanofluids	[70]
Chitin	Deep eutectic solvent method with simple heating	20.5	Green ^b^	8.9	380	~510	Fe^3+^ detection	[71]
Chitin (nanofibers)	Microwave assisted-hydrothermal	2–12	Blue ^b^	5.1	370	480	DPA sensing	[66]
Chitosan (85% DD)	Hydrothermal carbonization	3 ^c^	Yellow-brown ^a^, Blue ^b^	–	350	400	–	[66]
Chitosan (85% DA)	Hydrothermal carbonization	1.2–3.6	Yellowish transparent ^a^, Blue ^b^	9.3	340	~395	Fe^3+^, Ag ^+^ sensing	[75]
Chitosan (85% DA)	Hydrothermal carbonization	1–1.8	Yellowish transparent ^a^, Blue ^b^	15.3	340	~415	Fe^3+^, Ag ^+^ sensing	[75]
Chitosan	Hydrothermal carbonization	4–11	Blue ^b^	16.81	330	408	Cr (IV) sensing	[27]
Chitosan	Hydrothermal carbonization	1.5–3.1	Blue ^b^	–	290	412	PO_4_^3−^ sensing, bioimaging	[24]
Chitosan	Hydrothermal carbonization	0.5–4	Brown ^a^, Blue ^b^	38	457	533	Trace water detection	[76]
Chitosan (80–95% DD)	Hydrothermal carbonization	1–3	Blue ^b^	28.32	340	420	Packaging	[31]
Chitosan	Hydrothermal carbonization	3	Brown-yellow ^a^, Green ^b^	19	390	520	Enrofloxacin, NO_2_^−^ sensing	[74]
Chitosan (75% DD)	Hydrothermal carbonization	3–6	Blue ^b^	–	320	~405	Organophosphorus herbicide glyphosate sensing	[77]
Chitosan (91% DD)	Hydrothermal carbonization	2.6–5	Yellow ^a^, Blue ^b^	31.8	360	~440	Hg ^2+^ sensing	[72]
Chitosan (DD ≥ 95%)	Hydrothermal carbonization	2–10	Yellow ^a^, Blue ^b^	6.6	310	418	Fe^3+^ detection	[78]
Chitosan	Hydrothermal carbonization	6	Dark brown ^a^, Blueb	4.36	380	340	Solid-state emission component	[79]
Chitosan	Hydrothermal carbonization	4.02	Yellow ^b^	40	430	513	Solid-state emission component	[80]
Chitosan	Solvothermal	4	Blue ^b^	10.4	360	~450	Bioanalytical and bioimaging	[81]
Chitosan (85% DD)	Carbonization	3 ^c^	–	–	510	550	Drug delivery	[73]
Chitosan	Carbonization	1–6	Transparent ^a^, Blue ^b^	4.34	310	390	Cellular imaging	[82]
Chitosan	Thermal + freeze drying + milling	2–6	Blue ^b^	–	340	432	Fe^3+^ sensing	[83]
Chitosan (77.7% DD)	Microwave	2.7 ^c^	Yellow ^a^, Blue ^b^	–	360	~410	Detection of heavy metal ions	[84]
Chitosan	Microwave	0.6–8.7 ^c^	Yellow ^a^, Blue ^b^	–	300	334	–	[85]
Chitosan	Microwave heating method	3–4.8	Blue ^b^	25	350	450	Sensor for water detection in organic solvents	[29]
Chitosan	Microwave pyrolysis	2.7–6.5	Yellow-brown ^a^	6.4	338	440	–	[86]
Chitosan (DD ≥ 95%)	Microwave assisted-hydrothermal	4.8	Brown-yellow ^a^, Green ^b^	27	460	502	Sensing, security, and energy storage	[43]
Chitosan (85% DA)	Acid dehydration	1–2	Yellowish transparent ^a^, Blue ^b^	3.3	340	~405	Fe^3+^, Ag^+^ sensing	[75]
Chitosan quaternary ammonium salt	Hydrothermal carbonization	1.74	Yellow ^a^, Blue ^b^	9	340	~460	Visual treatment of bacterial infection	[87]
***Fish***								
Chondroitin sulfate (shark cartilage)	Hydrothermal carbonization	19.6–60	Yellow-brown ^a^, Yellowish-green ^b^, Blue ^b^	20.46	390	490	Bioimaging	[41]
Collagen and chitin (fish scales)	Hydrothermal carbonization	13	–	17.3	372	450	–	[88]
***Mixtures***								
Chitosan and κ-Carrageenan	Hydrothermal carbonization	8	Yellow ^a^, Blue ^b^	59.31	365	440	Fe^3+^, AA sensing	[100]
Chitosan and silk fibroin	Hydrothermal carbonization	1.5–4.5	Yellow ^a^, Blue ^b^	39–66	350	430	5-FU drug delivery	[101]
Chitosan and gum tragacanth	Hydrothermal carbonization	20	–	–	317	413	Bioimaging	[102]
Chitosan and acrylamide	Hydrothermal carbonization	1–3	Blue ^b^	–	360	438	Osteolytic diseases	[93]
Chitosan and acrylamide	Microwave-assisted-hydrothermal synthesis	–	Blue ^b^	12.17	330	409	Fe^2+^ detection	[46]

FL, fluorescence; AA, ascorbic acid; 5-FU, 5-fluorouracil; CdS, cadmium sulfide; DPA, D-penicillamine; DD, degree of deacetylation; DA, degree of acetylation; ^a^, under daylight; ^b^, under UV lamp; ^c^, hydrodynamic size obtained by dynamic light scattering.

## Data Availability

Not applicable.
